# Elevated Plasma Total Cholesterol Level Is Associated with the Risk of Asymptomatic Intracranial Arterial Stenosis

**DOI:** 10.1371/journal.pone.0101232

**Published:** 2014-07-03

**Authors:** Yuan Shen, Jing Wang, Jianwei Wu, Weikai Qu, Chunxue Wang, Xiang Gao, Yong Zhou, Anxin Wang, Shouling Wu, Xingquan Zhao

**Affiliations:** 1 Department of Neurology, Beijing Tiantan Hospital, Capital Medical University, Beijing, China; 2 Department of Surgery, The University of Toledo Medical Center, Toledo, Ohio, United States of America; 3 Channing Laboratory, Department of Medicine, Brigham and Women’s Hospital, Harvard Medical School, Boston, Massachusetts, United States of America; 4 Department of Nutrition, Harvard University School of Public Health, Boston, Massachusetts, United States of America; 5 Department of Cardiology, Kailuan Hospital, Hebei United University, Tangshan, China; University of Milan, Italy

## Abstract

**Background:**

Intracranial arterial stenosis (ICAS) is one of the most common causes of stroke, and dyslipidemia was one of the most common risk factors related to ICAS. However, the correlation between the plasma total cholesterol level (PTC) and ICAS, especially asymptomatic ICAS (AICAS) is not clear.

**Materials and Methods:**

5,300 participants were enrolled in this study. The diagnosis of AICAS was made by transcranial Doppler ultrasonography. The participants were then divided into 5 essentially equal-sized groups based on their PTC levels. The multivariate logistic regression was used to analyze the correlation between the PTC level and the prevalence of AICAS.

**Results:**

13.0% of the participants were diagnosed with AICAS. The prevalence of AICAS gradually increased with the increasing PTC level. After adjusted by the possible confounding factors, the Odds Ratios (OR) of the AICAS prevalence between the 1st quintile group and the other 4 groups were 1.13, 1.23, 1.63 and 1.75 with 95% confident intervals (CI) of 0.84–1.52, 0.91–1.66, 1.20–2.22 and 1.23–2.47, respectively. The further subgroup analysis revealed that the PTC level was stronger for males (OR 1.42 95%CI 1.23–1.64), regarding the prevalence of AICAS.

**Conclusions:**

In this large community-based study, the prevalence of AICAS is 13.0%, subjects with higher PTC levels showed a mild increase in the prevalence of AICAS. The PTC level is an independent risk factor of AICAS. Males seem to be significantly more vulnerable to the risk of AICAS.

## Introduction

Intracranial arterial stenosis (ICAS) is a common cause of ischemic stroke. 8.8% of the transient ischemic attack (TIA) and 3.7% of the ischemic stroke (IS) are caused by ICAS [1], [2]. The 1-year recurrence rate of TIA or IS is 23% in patients with ICAS of more than 70% [3]. In China, ICAS accounts for 33–50% of stroke and >50% of TIA, that is, the prevalence of ICAS is even higher than that of the Western population [4]–[6]. Therefore, it is important to screen for the risk factors of asymptomatic intracranial arterial stenosis (AICAS) and prevent the progress of AICAS through early interventions and decrease the risk of stroke, even death, in these patients.

The Warfarin-Aspirin Symptomatic Intracranial Disease (WASID) trial found that dyslipidemia was one of the most common risk factors related to symptomatic ICAS [7], and one of our studies demonstrated that non-high-density-lipoprotein-cholesterol and low density cholesterol were associated with AICAS [8]. Whether there is a relationship between the plasma total cholesterol (PTC) level, which is also an important part in lipid, and AICAS has not been thoroughly studied. Based on the Asymptomatic Polyvascular Abnormalities Community (APAC) study, we sought to analyze the correlation between the PTC level and the prevalence of AICAS in adult Chinese population.

## Methods

### Population and study design

The APAC study is a community-based, prospective, long-term follow-up observational study, to investigate the epidemiology of asymptomatic polyvascular abnormalities in Chinese adults. It is part of Kailuan study, which has been described previously [9]. A sample of 7000 subjects ≥40 years of age was randomly selected from Kailuan cohort, using stratified random sampling by age and sex, based on the data of state census in 2010. The sample size was calculated based on detection of 7% of event rate with 0.7% precision, α = 0.05. The response rate was assumed to be >80%. An initial cohort of 5,852 subjects participated the study, and 5,816 completed baseline survey and assessment from June 2010 to June 2011. Among the 5,816 individuals, 376 subjects were excluded following exclusion criteria (1) history of stroke, transient ischemic attack, and coronary disease at baseline; (2) presence of neurologic deficits which was estimated by experienced doctors. Finally, a total of 5,440 participants were eligible and included in APAC study. During the baseline survey, all the participants had undergone questionnaire assessment, clinical, laboratory, and transcranial Doppler (TCD) examinations. We excluded 65 participants who had incomplete data and 75 participants under lipid-lowering treatment (34 participants were treated with statins, 3 participants with fibrates, 36 participants with traditional Chinese medicine/others, 1 participant with statins and fibrates, another one were treated with statins and traditional Chinese medicine/others), leaving 3,186 men and 2,114 women in the analyses. There was no significant difference for the basic characteristics between the enrolled participants and those who were excluded (p>0.05, data not shown). The APAC study was performed according to the guidelines of Helsinki Declaration and was approved by the Ethics Committees of the Kailuan General Hospital and Beijing Tiantan Hospital. Written informed consent was obtained from all participants and approved by the above ethics committees.

### History and physical exam

A questionnaire was used to obtain baseline information, including age, sex, menopausal status, smoking, family history of cardiovascular disease, past medical history, such as hypertension, diabetes, hyperlipidemia, and medications prescribed by physicians. Weight, height and blood pressure (Bp) were measured during the baseline interview, and body mass index (BMI) was calculated. Bp was the average of two readings at rest. If the two measurements differed by more than 5 mm Hg, then an additional reading was taken, and the average of the three readings was used. We further categorized the subjects according to different parameters, i.e. age (<60 years and ≥60 years), BMI (<30 Kg/m^2^ and ≥30 Kg/m^2^), smoking status (current smoker who smokes at least one cigarette per day and non-smoker).

### Lab test

Blood samples were drawn from the antecubital vein in the morning after overnight fast. Tubes were centrifuged at 3000 ×g for 10 min at room temperature. After separation, plasma samples were frozen as rapidly as possible to −80°C for storage until laboratory determinations were performed. For all participants, PTC, high density lipoprotein cholesterol (HDL-C), low density lipoprotein cholesterol (LDL-C), triglycerides (TG) and fasting blood glucose (FBG) levels were assessed. All the blood variables were measured using an autoanalyzer (Hitachi 747; Hitachi, Tokyo, Japan) at the central laboratory of the Kailuan hospital. The participants were assigned into 5 quintile groups by their PTC level in ascending order.

### Definition of hypertension and diabetes

Hypertension was defined as: (1) previous history of hypertension diagnosed by a physician; (2) A systolic blood pressure (SBP) ≥140 mmHg, and/or a diastolic blood pressure (DBP) ≥90 mmHg; or (3) currently underwent antihypertensive treatment prescribed by a physician. Diabetes mellitus was diagnosed if (1) the subject was undergoing treatment with insulin or oral hypoglycemic agents; (2) FBG levels were >7.0 mmol/L; or (3) previous history of diabetes mellitus which was diagnosed by a physician.

### Transcranial Doppler Ultrasonography (TCD) Examination

TCD is a noninvasive, effective and reliable method to diagnose intracranial arterial stenosis [10], and it is easy to apply to a large groups of people, so we selected TCD as the method of choice to measure arterial stenosis in our study. The TCD exam was performed by two experienced radiologist using a portable devices (EME Companion, Nicolet). A diagnosis of AICAS was established following peak systolic flow velocity (PSV) criteria that were validated against MR angiography and clinical outcomes [11], [12]: >140 cm/second for the middle cerebral artery (MCA); >120 cm/second for the anterior cerebral artery (ACA); >100 cm/second for the posterior cerebral (PCA), vertebral and basilar arteries; and >120 cm/second for the internal carotid siphon (SIPH). In addition to the PSV criteria, the participants’ age, disturbance of the echo frequency, turbulence or abnormal blood flow were also taken into consideration for AICAS diagnosis [11]. AICAS was diagnosed if at least one of the studied arteries showed evidence of stenosis. Undetected arteries via both temporal and orbital window were considered negative for ICAS [11].

### Statistical analysis

Statistical analysis was performed using SAS software, ver. 9.1 (SAS Institute, Cary, North Carolina, USA). As all the continuous variables were in skewed distribution, medians were used for analysis. Comparison of continuous data was done by Analysis of Variance (ANOVA) and categorical data was done by Chi-square tests. Logistic regression models were used to calculate odd ratios (OR) of the effects of different PTC levels on the presence of AICAS. The variables adjusted for were age, sex, hypertension, diabetes, smoking, family history of myocardial infarction (MI) and stroke, BMI, SBP, DBP, FBG, LDL-C, HDL-C,TG and anti-hypertension treatment. Additionally, the relationship between different PTC levels and AICAS was further analysed in subgroups, such as age, sex, menopause, hypertension, diabetes, smoking and BMI. As P interaction for sex and PTC level was <0.05, we also added the interaction term into the main logistic model. All statistical analyses were two-tailed, and a P-value less than 0.05 was considered statistically significant.

## Results

### Prevalence of AICAS

**Figure 1:**
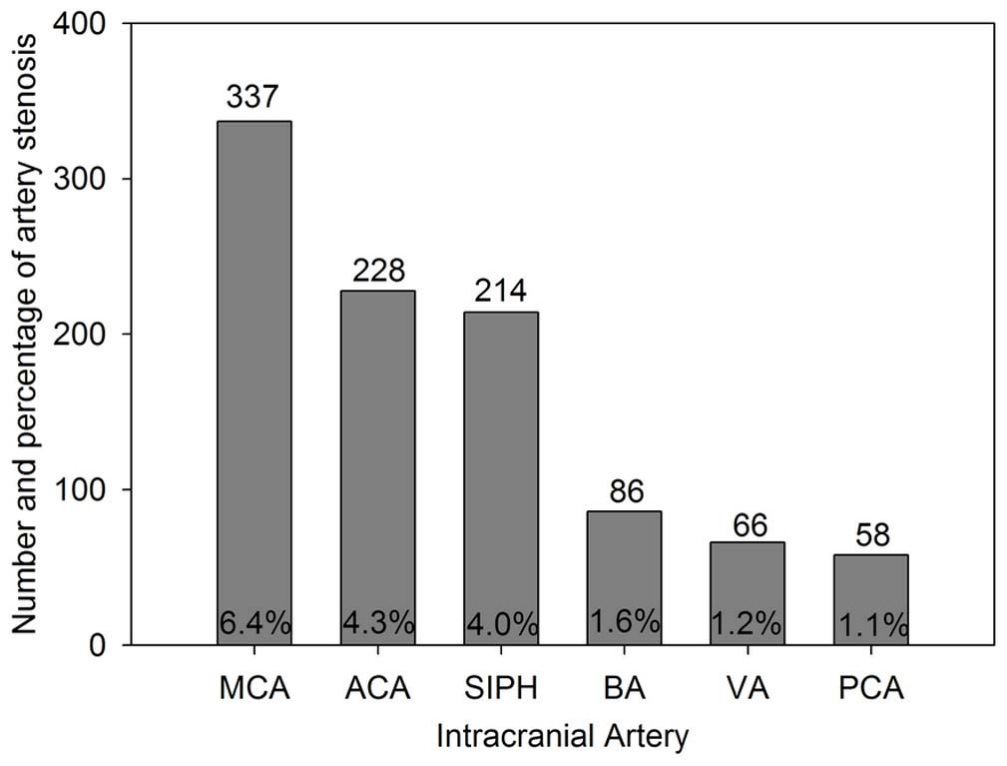
The incidence of stenosis in different intracranial arteries. ACA: anterior cerebral artery; BA: basal artery; MCA: middle cerebral artery; PCA: posterior cerebral artery; SIPH: siphon carotid artery; VA: vetebral artery.

In our study, there were 460 subjects (female, 150 subjects, accounts for 32.6%) who could not be fully evaluated, which means those have at least one undetected artery. 691 (13.0%) of the participants were diagnosed of AICAS by TCD. The lesion affected a variety of the intracranial arteries (Fig. 1). There were 336 participants (6.3%) with stenosis in a single artery, 178 participants (3.4%) in two arteries, and 177 participants (3.3%) in more than 2 arteries.

### Baseline Characteristics

The baseline characteristics of each quintile PTC group were shown in Table 1. The median and range of the PTC level in different groups were 3.91 mmol/L (≤4.23 mmol/l), 4.49 mmol/l (4.24–4.72 mol/l), 4.95 mmol/l (4.73–5.19 mmol/l), 5.49 mmol/l (5.20–5.80 mmol/l) and 6.32 mmol/l (≥5.81 mmol/l), respectively. As the PTC levels increased from the 1^st^ quintile to the 5^th^ quintile group, the medians of FBG, LDL-C, HDL-C and TG level were increased across the quintile PTC groups. Family history of MI, participants with hypertension and anti-hypertension treatment, diabetes or obesity were more prevalent in the groups with higher PTC levels.

**Table 1:** Baseline characteristics of participants in different PTC quintile groups.

	PTC levels, quintile groups					
	Q1	Q2	Q3	Q4	Q5	P value
Number	1060	1069	1069	1039	1063	
PTC, median(Q1–Q4), mmol/l	3.91(3.67–4.10)	4.49(4.36–4.61)	4.95(4.83–5.07)	5.49(5.34–5.62)	6.32(6.02–6.82)	<0.001
Age, median(Q1–Q4), years	50.51(44.25–60.92)	51.19(44.84–59.44)	52.32(45.73–61.00)	53.39(46.33–61.83)	54.17(47.16–66.10)	<0.001
Women,n(%)	421(39.7)	427(39.9)	420(39.3)	417(40.1)	429(40.4)	0.99
Postmenopausal females,n(%)	157(37.3)	177(41.5)	218(51.9)	240(57.6)	299(69.7)	0.30
Smoking,n(%)	324(30.6)	328(30.7)	351(32.8)	327(31.5)	369(34.7)	0.21
Hypertension,n(%)	466(44.0)	465(43.5)	509(47.6)	527(50.7)	568(53.4)	<0.001
Diabetes,n(%)	91(8.6)	101(9.4)	138(12.9)	136(13.1)	165(15.5)	<0.001
Family history of MI,n,%	49(4.6)	54(5.1)	67(6.3)	67(6.4)	96(9.0)	0.001
Family history of stroke,n(%)	191(18.0)	185(17.3)	179(16.7)	196(18.9)	200(18.8)	0.54
BMI,≥30 Kg/m^2^,n(%)	462(43.6)	476(44.5)	486(45.5)	499(48.0)	541(50.9)	0.005
SBP, median(Q1–Q4), mmHg	129.33(116.00–140.00)	129.33(116.00–140.00)	130.00(120.00–141.33)	130.00(120.00–143.33)	130.00(120.00–146.00)	<0.001
DBP, median(Q1–Q4), mmHg	80.00(73.75–90.00)	80.00(75.00–90.00)	80.67(76.67–90.00)	80.67(78.67–90.00)	80.67(77.33–90.00)	0.001
FBG, median(Q1–Q4), mmol/L	5.07(4.69–5.51)	5.09(4.73–5.60)	5.20(4.84–5.86)	5.30(4.88–5.87)	5.40(4.98–6.06)	<0.001
LDL-C, median(Q1–Q4), mmol/l	2.08(1.72–2.40)	2.40(2.07–2.74)	2.61(2.30–2.94)	2.85(2.50–3.25)	3.25(2.82–3.78)	<0.001
HDL-C, median(Q1–Q4), mmol/l	1.43(1.22–1.70)	1.50(1.29–1.81)	1.58(1.30–1.86)	1.63(1.35–1.98)	1.75(1.45–2.11)	<0.001
TG, median(Q1–Q4), mmol/l	1.06(0.77–1.51)	1.19(0.86–1.61)	1.24(0.94–1.79)	1.46(1.06–2.10)	1.72(1.14–2.55)	<0.001
Anti-hypertension treatment,n(%)	174(16.4)	168(15.7)	200(18.7)	229(22.0)	233(21.9)	<0.001

BMI: body mass index; DBP: diastolic blood pressure; FBG: fasting blood glucose; HDL-C: high-density lipoprotein cholesterol; LDL-C: low-density lipoprotein cholesterol; MI: myocardial infarction; PTC: plasma total cholesterol; SBP: systolic blood pressure; TG: triglycerides.

### Correlation between PTC level and AICAS

**Figure 2:**
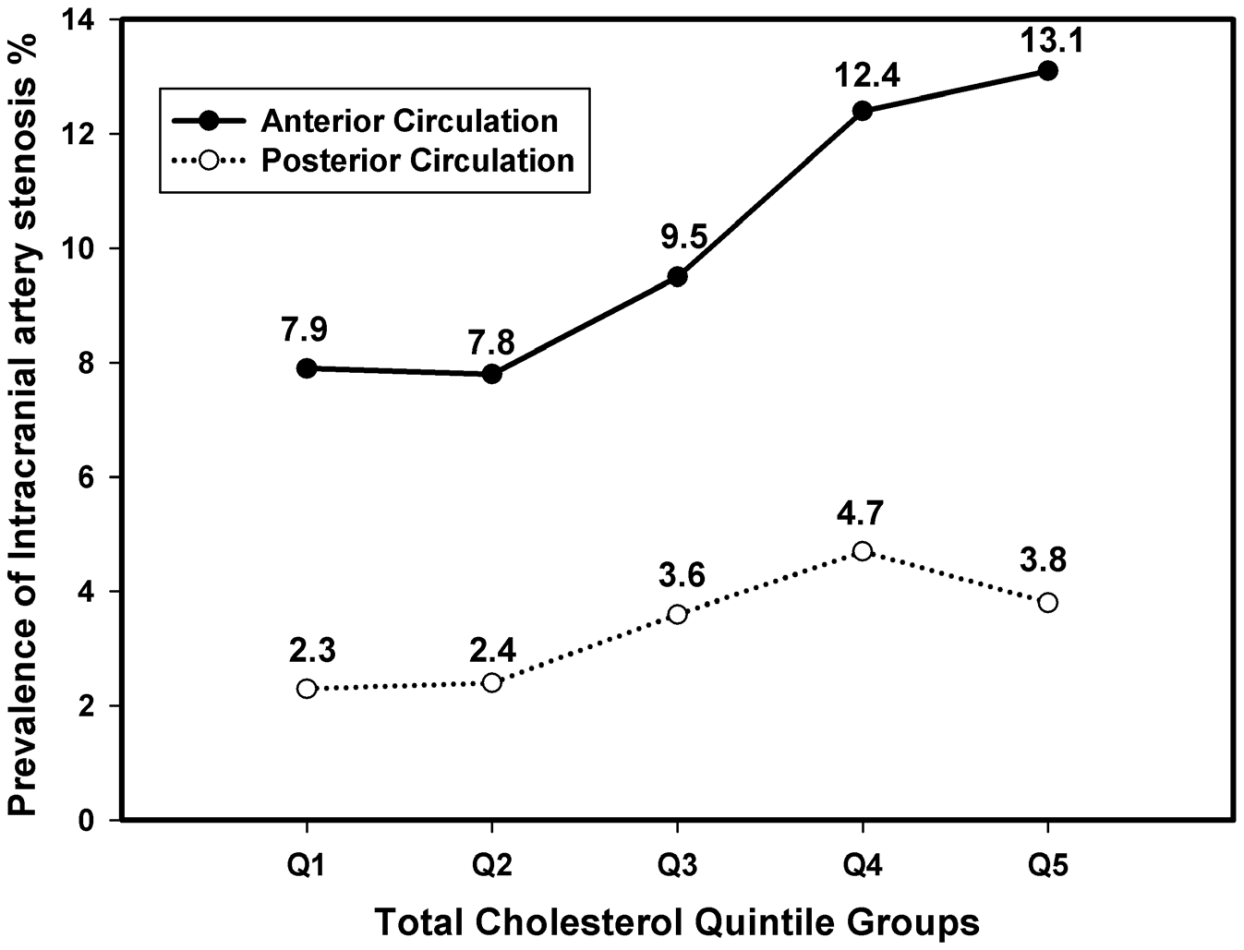
The prevalence of AICAS in each PTC quintile group. AICAS: asymptomatic intracranial artery stenosis; PTC: plasma total cholesterol.

**Figure 3:**
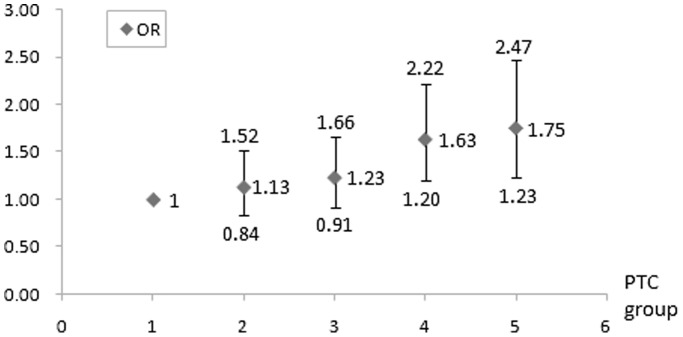
ORs and 95%CIs for AICAS between different PTC quintile groups. OR: odd ratio; CI: confidential interval; AICAS: asymptomatic intracranial artery stenosis; PTC: plasma total cholesterol.

In order to show the correlation between PTC level and the prevalence of AICAS clearly, MCA, ACA and SIPH were considered as anterior circulation, BA, VA and PCA were considered as posterior circulation, and Fig. 2 shows that the prevalence of AICAS in the anterior and posterior circulation increases with the PTC level rising. PTC level was proven to be an independent risk factor of AICAS by both un-adjusted and adjusted logistic regression analysis in our study (crude OR 1.24, 95%CI 1.15–1.34; adjusted OR 1.28, 95% CI 1.14–1.43). After adjusted by age, sex, hypertension, diabetes, smoking, family history of MI and stroke, anti-hypertension treatment, BMI, SBP, DBP, FBG, LDL-C, HDL-C and TG, the prevalences of AICAS in the 4^th^ and 5^th^ quintile group were significantly higher than that of the 1^st^ quintile PTC group (4^th^ vs. 1^st^ OR 1.63, 95% CI 1.20–2.22; 5^th^ vs. 1^st^ OR 1.75, 95%CI 1.23–2.47). (Table 2 and Fig. 3).

**Table 2:** Odd ratios (OR) for AICAS between different PTC quintile groups.

	PTC levels					
	Q1	Q2	Q3	Q4	Q5	Continuous Scale
AICAS N(%)	108(10.2)	113(10.6)	132(12.3)	163(15.7)	175(16.5)	691(13.0)
Crude OR (95%CI)	1	1.04(0.79–1.38)	1.24(0.95–1.63)	1.64(1.27–2.13)	1.74(1.34–2.25)	1.24(1.15–1.34)
Model 1 OR (95%CI)	1	1.07(0.81–1.42)	1.24(0.94–1.63)	1.61(1.24–2.10)	1.62(1.25–2.10)	1.19(1.10–1.29)
Model 2 OR, 95%CI	1	1.13(0.84–1.52)	1.23(0.91–1.66)	1.63(1.20–2.22)*	1.75(1.23–2.47)*	1.28(1.14–1.43)**

* P<0.05;

** P<0.001.

95%CI: 95% confidence interval; AICAS: asymptomatic intracranial artery stenosis; MI: myocardial infarction; PTC: plasma total cholesterol.

PTC levels: Q1≤4.23 mmol/l, Q2 4.24–4.72 mol/l, Q3 4.73–5.19 mmol/l, Q4 5.20–5.80 mmol/l and Q5≥5.81 mmol/l.

Model 1: adjusted for age, sex;

Model 2: adjusted for age, sex, hypertension, diabetes, smoking, family history of myocardial infarction and stroke, BMI, SBP, DBP, FBG, LDL-C, HDL-C, TG, anti-hypertension treatment and sex*PTC.

**Figure 4:**
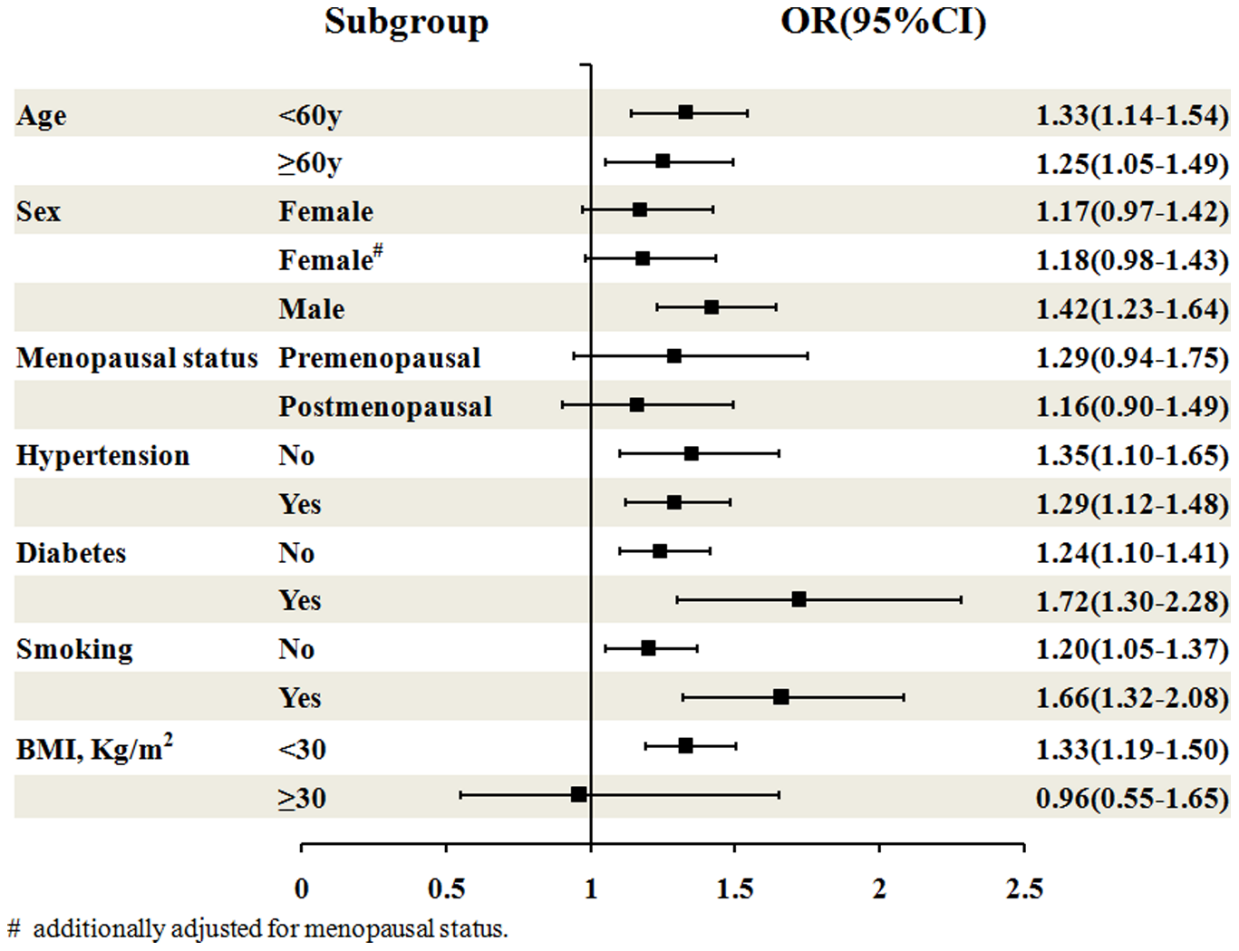
ORs and 95%CIs for AICAS of PTC level in different subgroups. # additionally adjusted for menopausal status. OR: odd ratio; CI: confidential interval; AICAS: asymptomatic intracranial artery stenosis; PTC: plasma total cholesterol.

Further analyses of the interaction effects on the association between PTC levels and the prevalence of AICAS showed that there was a significant difference between men and women (P for interaction, 0.001), and that the association was statistically significant in men only (P<0.05), Our results indicated that in men, the prevalence of AICAS was significantly increased with increasing PTC levels (OR 1.42, 95%CI: 1.23–1.64), especially when PTC ≥4.73 mmol/L (Q3 OR 1.70 95%CI 1.14–2.55; Q4 OR 2.41 95%CI 1.59–3.64; Q5 OR 3.10 95%CI 1.95–4.91). In contrast, in women, PTC levels were not an independent indicator for the presence of AICAS (OR 1.17, 95%CI: 0.97–1.42). When other baseline characteristics (including age, menopausal status, hypertension, diabetes, smoking and BMI) were evaluated, the presence or absence of these indicators did not influence the association between PTC levels and the prevalence of AICAS (P = 0.32, 0.42, 0.94, 0.46, 0.16 and 0.15 respectively), although the OR values for some subgroups were significant (Table 3 and Fig. 4).

**Table 3:** Multivariate-adjusted odd ratios (OR) for AICAS between different PTC quintile groups, stratified by age, sex and selected risk factors.

	PTC levels, quintile groups						
	Q1	Q2	Q3	Q4	Q5	Continuous Scale (1 mmol/L increment)	P interaction
Age							0.32
<60 y	1	0.96(0.66–1.40)	1.03(0.70–1.51)	1.80(1.22–2.66)*	1.52(0.96–2.41)	1.33(1.14–1.54)**	
≥60 y	1	1.57(0.98–2.54)	1.83(1.13–2.96)*	1.64(1.00–2.70)	2.45(1.42–4.22)*	1.25(1.05–1.49)*	
Sex							0.001
Female	1	0.84(0.54–1.33)	0.94(0.60–1.49)	1.16(0.73–1.86)	0.98(0.56–1.70)	1.17(0.97–1.42)	
Female^#^	1	0.85(0.54–1.34)	0.94(0.60–1.49)	1.19(0.74–1.90)	1.01(0.58–1.76)	1.18(0.98–1.43)	
Male	1	1.47(0.99–2.18)	1.70(1.14–2.55)*	2.41(1.59–3.64)**	3.10(1.95–4.91)**	1.42(1.23–1.64)**	
Menopausal status							0.42
Premenopausal	1	0.71(0.39–1.31)	0.80(0.42–1.53)	1.02(0.50–2.05)	1.17(0.49–2.80)	1.29(0.94–1.75)	
Postmenopausal	1	1.14(0.55–2.35)	1.18(0.59–2.35)	1.36(0.68–2.73)	1.02(0.47–2.24)	1.16(0.90–1.49)	
Hypertension							0.94
No	1	0.98(0.62–1.56)	1.05(0.65–1.71)	1.29(0.77–2.16)	1.90(1.08–3.33)*	1.35(1.10–1.65)*	
Yes	1	1.30(0.88–1.91)	1.43(0.98–2.10)	2.04(1.38–3.01)**	1.92(1.23–3.00)*	1.29(1.12–1.48)**	
Diabetes							0.46
No	1	1.14(0.83–1.56)	1.16(0.84–1.61)	1.48(1.06–2.07)*	1.54(1.05–2.26)*	1.24(1.10–1.41)*	
Yes	1	1.36(0.57–3.24)	2.33(1.04–5.22)*	4.19(1.85–9.51)*	6.32(2.45–16.31)**	1.72(1.30–2.28)**	
Smoking							0.16
No	1	1.20(0.85–1.69)	1.26(0.89–1.80)	1.46(1.02–2.11)*	1.43(0.94–2.17)	1.20(1.05–1.37)*	
Yes	1	1.06(0.59–1.92)	1.37(0.77–2.43)	2.90(1.62–5.20)**	3.67(1.91–7.03)**	1.66(1.32–2.08)**	
BMI, Kg/m^2^							0.15
<30	1	1.13(0.83–1.53)	1.27(0.93–1.73)	1.72(1.25–2.36)*	1.97(1.37–2.82)**	1.33(1.19–1.50)**	
≥30	1	1.50(0.39–5.77)	1.10(0.28–4.38)	2.18(0.54–8.83)	0.92(0.18–4.82)	0.96(0.55–1.65)	

* P<0.05; ** P<0.001.

AICAS: asymptomatic intracranial artery stenosis; BMI: body mass index; PTC: plasma total cholesterol.

PTC levels: Q1≤4.23 mmol/l, Q2 4.24–4.72 mol/l, Q3 4.73–5.19 mmol/l, Q4 5.20–5.80 mmol/l and Q5≥5.81 mmol/l.

Multivariate-adjusted odd ratios (OR): adjusted for age, sex, hypertension, diabetes, smoking, family history of myocardial infarction and stroke, BMI, SBP, DBP, FBG, LDL-C, HDL-C, TG, anti-hypertension treatment and sex*PTC.

# additionally adjusted for menopausal status.

## Discussions

ICAS prevention is considered one of the most important measures to reduce the risk of cerbral vascular accident. Previous studies have shown that ICAS was closely related to the occurrence of ischemic stroke [13]. In a group of participants who were followed up for a median of 18 months, researchers found that 18.6% of all symptomatic ICAS patients would eventually develop ischemic stroke and 13.2% of the ischemic stroke patients were caused by AICAS [13]. Similar results were also seen in Chinese population. One study contained 705 Chinese patients with acute ischemic stroke and followed up for 42 months, eventually it was found that the annual recurrence rates of stroke caused by ICAS in the 1^st^ and 2^nd^ year during follow-up were 17.1% and 8.6%, respectively [6].

Since the PTC level had been shown to be positively correlated with the incidence of ischemic stroke [14], we sought to investigate the relationship between the PTC level and the prevalence of AICAS in this study. Our results showed that the risk of AICAS was related to the PTC level. It has been proven that dyslipidemia was strongly associated with the severity of ICAS [7], [15]. In the Warfarin-Aspirin Symptomatic Intracranial Disease (WASID) trial, dyslipidemia was found to be one of the most common risk factors related to severe ICAS [7]. Investigation in a group of 1,471 TIA and minor stroke patients revealed that the low HDL-C level coupled with high TG level was strongly related to the occurrence of symptomatic ICAS and the risk of early stroke was significantly higher in the patients with hyperlipidemia than in the patients without this disorder [15]. Our previous study showed that non-HDL was associated with ICAS [8], and concentrations of apolipoprotein E [16] and lipoprotein(a) [17] were also found to be risk factors for ICAS. However, current evidences of the correlation between PTC and ICAS, especially with AICAS were limited.

The prevalence of AICAS was 11.8% (250 cases) in females and 13.8% (441 cases) in males in our study. We also found that the elevated PTC level was significantly related to the risk of AICAS in male participants but not in females. This finding was in agreement with the results from previous studies [2], [18]. Several factors might contribute to this phenomenon. First, sex hormones might play an important role in this process [18]. Cholesterol is an important precursor for the synthesis of steroid hormones, including the sex hormones progesterone, estrogens and testosterone. There were evidences shown that the hormone replacement therapy in postmenopausal women could significantly decrease the risk of atherosclerosis [19]. There were 1,091 (51.6%) postmenopausal females in our study. The elevated PTC level might have some protective effect to prevent them from developing atherosclerosis, which is the most important cause of ICAS. Second, the efficacy of the TCD decreased with age and the trend were more commonly seen in women than in men [20]. Since all the participants with a poor temporal window reading were considered without AICAS, this might result in disproportionally underestimating the prevalence of AICAS in the females. However, in our population, there is no significant difference of subjects with poor temporal window between male and female in age<60 y and ≥60 y groups (data not shown). The aim of our large-scale cross-sectional study was to investigate the possible correlation between PTC level and AICAS. There were some limitations in our study despite of the careful study design. First, all participants with poor temporal window reading in TCD were considered non-ICAS in our study. This might result in a significantly underestimated prevalence of ICAS. Second, all the participants were from area and same ethnic group. This would limit the application of the findings to a population with broader geographic and ethnic diversity. Third, although TCD has been regarded as a noninvasive and convenient screening method to diagnose ICAS, it is not as accurate as DSA for determining ICAS [21]. Despite these limitations, our study was so far the first large-scale clinic trial to our knowledge that investigate the correlation between PTC level and AICAS. We also excluded the patients who were taking antihyperlipidemic medicine at the recruitment to eliminate the drug interference to the baseline PTC level.

## Conclusions

PTC level was an independent risk factor for the occurrence of AICAS, especially in males. Further studies are needed to provide us more information on its treatment and prognosis.
